# Parameters for Change in Offline Gambling Behavior After the First COVID-19 Lockdown in Germany

**DOI:** 10.3389/fpsyg.2022.857234

**Published:** 2022-07-05

**Authors:** Jens Kalke, Christian Schütze, Harald Lahusen, Sven Buth

**Affiliations:** ^1^Department of Psychiatry, Centre for Interdisciplinary Addiction Research of Hamburg University, University Medical Centre Hamburg-Eppendorf, Hamburg, Germany; ^2^Institute for Interdisciplinary Addiction and Drug Research, Hamburg, Germany

**Keywords:** gambling, venues, closures, influencing factors, logistic regression, COVID-19

## Abstract

**Introduction:**

In spring 2020, the first nationwide lockdown in response to the spreading COVID-19 pandemic came into effect in Germany. From March to May, gambling venues, casinos, and betting offices were forced to close. This study explores how land-based gamblers respond to short-term closures of higher-risk forms of gambling. Which gamblers are particularly susceptible to switching to online gambling? Which are more likely to use the lockdown as an opportunity to quit or pause gambling? Potential parameters for these switching or cessation processes are identified using multivariate multinomial logistic regression analysis.

**Methods:**

The research questions are analyzed on the basis of quantitative data. For this purpose, a survey was conducted among members of a comparatively large German online access panel (“PAYBACK panel”). The sample of analysis consisted of 612 gamblers who had participated in at least one higher-risk form of gambling and had done so exclusively offline before the first lockdown (January and February 2020).

**Results:**

A total of 37.1% of sports bettors ceased participation in higher-risk forms of gambling, compared to 64.1% of casino gamblers. Switching to online gambling, on the other hand, was a rather rare event, regardless of the form of gambling: the proportions differ between 7.7% (slot machines) and 10.9% (sports betting). In the multivariate model female gender, younger age, and a lower frequency of gambling before the first lockdown were found to be significant factors for quitting (instead of continuing) higher-risk offline gambling forms. Secondly, the analysis revealed that individuals with more pronounced cognitive distortions had an increased risk of switching to online gambling rather than staying offline.

**Discussion:**

A key finding of this study is that the temporary closure of offline venues does not result in a significant shift towards the online market. Instead, the results of this study show that these short, temporary closures of gambling venues were an appropriate opportunity to give individual groups of gamblers the opportunity to reflect, reduce or quit gambling. It is worth considering implementing such temporary closures as a preventive measure in the future – this should be investigated in advance in further evaluation studies.

## Introduction

In Germany, the first nationwide lockdown in response to the spreading COVID-19 pandemic came into effect in mid-March 2020. As a result, only facilities and businesses of systemic importance or those which provided essential goods were allowed to remain open to the public, while some of the offline gambling venues were explicitly required to close. These regulations varied among the different gambling services: lottery retail outlets were allowed to remain open if the sale of gambling products was ancillary and the (main) business consisted in selling products for essential daily needs (newspaper sales, food, fuel, etc.). This was the case for most lottery retail outlets in Germany. On the other hand, slot machine venues, casinos (state-licensed businesses), and betting shops had to close and were not allowed to receive customers. During the lockdown, violations of these regulations occurred and thus illegal offline gambling took place (slot machines or betting opportunities available to the public). If detected, these activities were terminated by municipal public order offices, police, and public prosecutors. In May and June 2020, the pandemic-related closures of offline gambling venues were again gradually rolled back by the federal states. Nevertheless, offline gambling venues had to comply with certain hygiene requirements: a maximum number of customers per venue, physical distancing rules, and mandatory face masks. Operators were obliged to compile and implement hygiene concepts. Among other things, partitions had to be set up between vending machines, live broadcasting of sports events was prohibited, consumption of food was forbidden, ventilation and cleaning had to be warranted, and customer compliance with the rules had to be ensured [Corona ordinances of the federal states, e.g., Hamburg (Senat der Freien und Hansestadt Hamburg, 2020)].

In 2019, the volume of the German gambling market was estimated at a total of 13.3 billion euros in net gambling losses (wagers minus wins). This includes online and offline, as well as legal and illegal services (hardly any of the sports betting services operating in Germany in 2019 had the required official permission, online casino games and online slot machines were not eligible for permission) (Glücksspielaufsichtsbehörden der Länder, 2020). Among offline services, 3.3 billion euros were attributable to the lotteries of the 16 state lottery companies with approximately 21,200 retail outlets, 5.5 billion euros to approximately 5,000 slot machine operators (slot machine venues, restaurants, and bars), and 860 million euros to 70 casinos. For the – mostly illegal – sports betting services with their 4,000–5,000 offline gambling venues, the net gambling losses are estimated at about 900 million euros. Therefore, the offline gambling services with the highest risk potential (casinos, slot machines, and sports betting) represent about 55% of the entire German gambling market with a total of 7.26 billion euros in net losses. In the first year of the pandemic, the summarized net gambling losses fell to 11.7 billion euros (Glücksspielaufsichtsbehörden der Länder, 2021). Online gambling accounted for around 19% of the net gambling losses in 2020, which was around 4% points more than in the previous year. Until then, there was no consistent development: the share of online gambling had increased from 14 to 21% from 2014 to 2017, but then decreased again until 2019 (Glücksspielaufsichtsbehörden der Länder, 2015, 2016, 2017, 2018, 2019, 2020, 2021).

In 2019, 35% of the population in Germany participated in gambling at least once: 21% took part in the “6aus49” number lottery, 9% used scratch cards, 3% slot machines, 2% sports betting, and 1.5% participated in casino games (slot machines, card games, and roulette) (Banz, 2020). With regard to gambling related problems, the representative monitoring of the gambling behavior among the German general population reports 0.39% problem gamblers and 0.34% pathological gamblers for the year 2019. A total of 3.52% of the population were classified as at-risk gamblers (Banz, 2020).

In general, all studies on the influence of pandemic-related restrictions on gambling behavior report declines in participation (Hodgins and Stevens, 2021). However, some of the individual results of the analyses differ among studies, e.g., with respect to the extent of changes in gambling behavior or among subgroups of the analyses. This may be due to divergent gambling regimes or varying COVID-19 restrictions. In Canada, for example, almost all gambling venues were closed (Shaw et al., 2021), while in Australia poker and slot machines were closed nationally for 3 months (Gainsbury et al., 2021), and in Sweden where all casinos were closed, further efforts were undertaken to reduce the time spent on land-based gambling machines (as well as on online casino games) with mandatory limits and other regulations to reduce gambling participation (e.g., limit of deposits) (Hakansson, 2021). Furthermore, the methodological approaches of the studies varied. These diverse conditions make it difficult to formulate generalizing statements. In addition, only few available studies explicitly report on the change from offline to online participation or the cessation of gambling with regard to specific forms of gambling (e.g., slot machines) during the COVID-19 lockdowns. In contrast, statements on general changes in gambling behavior are found more frequently.

Switching to online gambling: in a United States study, Xuereb et al. (2021) report that 15% of participating gamblers had exclusively used offline gambling services before the lockdown and switched to online gambling during the lockdown. Similarly, in a Canadian study, 18% of offline-only gamblers switched to online gambling during the lockdown (Shaw et al., 2021). In New Zealand, however, the figure switching from offline to online gambling was lower (8%) (Hiringa, 2020). Those who switched featured higher proportions of people with gambling problems and with lower incomes than those who had never participated in online gambling (Xuereb et al., 2021). A study by Price (2022) showed that those gamblers who switched to online gambling had a significantly lower risk of exhibiting gambling disorders than those who had already engaged in online gambling before the lockdown (Price, 2022).

Gambling cessation: 28% of the gamblers in the Canadian study quit gambling during the lockdown (Shaw et al., 2021). Georgiadou et al. (2021) report that a total of 39% of the gamblers interviewed in Germany ceased their gambling activities during lockdown – among offline-only gamblers, the figure was 50%. In particular, gamblers who engaged in online sports betting (80%), offline slot machines (76%), and offline lotteries (55%) reduced or quit gambling altogether (Georgiadou et al., 2021). In contrast, a Swedish study reported much smaller reductions of offline gambling participation: 7% for offline sports betting, 8% for offline horse betting, 6% for offline slot machines, and 13% for offline lotteries (Hakansson, 2021). Cessation or reduction in gambling participation was statistically significantly associated with an age below 35 years and above 54 years, and a school attendance of at least 11 years. At the same time, decreases in gambling participation were also associated with increased perception of own pandemic-related stress (Georgiadou et al., 2021). In contrast, Biddle (2020) concludes for a sample of Australian gamblers that the largest reductions in gambling participation were found in both the middle age group of 35- to 45-year-olds and among individuals from regions with lowest socioeconomic indices. Gunstone et al. (2020) report that 22% of United Kingdom gamblers who reduced or quit gambling during lockdown did so because sporting events that otherwise could have been bet on had been canceled. This was true to a greater extent for men (34%) than for women (11%). This was also a significant motive for younger individuals and those with higher Problem Gambling Severity Index (PGSI) scores to reduce or cease gambling participation (Gunstone et al., 2020).

Similar to the research outlined above, the study at hand addresses the question of whether changes in gambling behavior have occurred during and following the initial pandemic-related lockdown. Furthermore, this study focusses exclusively on casino games, slot machines, and sports betting as these gambling forms are all available offline as well as online and because they all have a considerably elevated addiction potential (Meyer et al., 2010). This is exceedingly true for the online version of these gambling forms (Hayer et al., 2019). In general, online gambling is considered to be even more problematic compared to offline gambling, especially due to the possibility of cashless payment and anonymity (Gainsbury, 2015). A recent meta-analysis identified internet gambling as the strongest risk factor for developing problem gambling behavior (Allami et al., 2021). Offline gambling providers have therefore often argued that a (state-enforced) reduction in offline services would lead to a migration of offline gamblers toward online services, thus increasing the number of problem gamblers. The pandemic lockdowns provide an unexpected opportunity for academic researchers to test this hypothesis in an empirical manner.

The main focus of the present study is to identify potential parameters for a change of gambling behavior in the higher-risk gambling forms. The study’s specific research questions are as follows:

1. Which gamblers are particularly susceptible to switching toward online gambling?
2. And which gamblers are more likely to use the lockdown as an opportunity to quit or pause their gambling activities?

Potential influencing factors for these switching and withdrawal processes will be identified by means of multivariate logistic regression. Based on this specific research question, the present study will extend the existing body of research on the effects of lockdowns on gambling behavior.

## Materials and Methods

The research questions formulated above were to be analyzed on the basis of quantitative data. For this purpose – as for answering further research questions –, an online-survey was conducted among members of a comparatively large German online access panel (“PAYBACK online panel”) using the statistical survey web app “LimeSurvey.” The panel includes people who are generally willing to participate in online surveys on a wide range of topics. At the time of the survey – December 2020 and January 2021 – the panel comprised around 120,000 people.

As part of our study, the members of the panel received an invitation to participate in the survey, stratified by age, gender, and region of residence. The invitation did not contain any information that the study focused on gambling as to keep participants from knowingly providing false information on gambling participation. In total, 45,779 of the approximately 120,000 payback panel members accepted the invitation to participate in a short screening. Those who met the inclusion criteria (participation in gambling immediately before or during the phases of the pandemic in 2020) were asked to complete a longer questionnaire. After completing the questionnaire, the respondents received compensation in terms of payback points.

In order to draw conclusions on users of different forms of gambling, a sample with a sufficiently large number of cases for each gambling form (lotteries, scratch cards, casino games, slot machines, and sports betting) was needed. A further aim of the original study was to differentiate between specific gambling settings (predominantly online, predominantly offline, and online and offline). The target figure for each of these gambling groups was minimum 300 respondents. This ensures that sufficient cases are available for the analysis of specific questions (e.g., changes in gambling behavior due to the pandemic-related lockdown).

Once this number of *N* = 300 cases had been reached, persons in question were only included if they also reported participation in other forms of gambling which had not yet reached the quota. This was done in order to keep the costs of the survey within reasonable limits.

At the end of the survey, a total of 4,672 people had completed questionnaires (for all gambling forms and gambling settings). As formulated in the research questions above, only gamblers who had participated in at least one of the higher-risk forms of gambling and had done so exclusively offline before the first lockdown (January and February 2020) were included in the further analyses (*N* = 612). The target figure (*N* = 300 per group) could not be reached for casino games (*N* = 282) and sports betting (*N* = 280). Indication of multiple gambling forms (e.g., participation in slot machine and casino games in parallel) was possible.

The PGSI was used to assess gambling problems (Ferris and Wynne, 2001). The PGSI allows for classifying at-risk gambling behavior even below the threshold of disordered gambling (4 problem groups: non-problem gambling, low level of problems, moderate level of problems, and problem gambling). The PGSI possesses strong internal consistency (Cronbach’s alpha = 0.84) and according to Ferris and Wynne (2001), it correlates at *r* = 0.83 each with two other instruments commonly used to determine gambling problems (DSM-IV, SOGS). It contains nine questions which primarily refer to the consequences of problem gambling. The answer options and corresponding scores are: 0 = never; 1 = sometimes, 2 = most of the time, 3 = almost always. The scores for all answers are summed up to determine the extent of the problem. A sum score of eight points or more indicates an existing gambling problem.

The Alcohol Use Disorder Identification Test-Consumption (AUDIT-C) was used to assess risky drinking (Bush et al., 1998). It comprises three questions related to frequency and quantity of alcohol use. Respondents can score between 0 and 4 points for each answer, so that the maximum sum score is 12 points. The cut-off for risky drinking behavior is 4 points for women and 5 points for men. The internal consistency (Cronbach’s alpha) of the German language version is 0.56, its sensitivity is 0.74, and its specificity 0.83 (Rumpf et al., 2002).

The German-language version of the Mental Health Inventory-5 (MHI-5) (Berwick et al., 1991; Rumpf et al., 2001) was used as a screening instrument for mental health. It consists of five questions related to nervousness, feeling down without being able to cheer up, discouragement and sadness, serenity, and happiness in the last 4 weeks. Unlike the original English version, the German version contains only five (instead of 6) possible answers, ranging from “never” (1) to “always” (5). After reversing the polarity of the two items “serenity” and “happiness,” the scores for the individual questions are summed up and transformed so that the sum score can vary between 0 (extremely mentally impaired) and 100 (extremely mentally healthy). The MHI-5 was validated for Germany by Rumpf et al. (2001). In the receiver operated characteristics (ROC) curve analysis, an area under the curve (AUC) of 0.88 was found for affective disorders and 0.71 for anxiety disorders.

The Gamblers, Beliefs Questionnaire was developed by Steenbergh et al. (2002) to assess cognitive distortions with regard to gambling. It contains a total of 21 statements which are rated on a scale from 0 (strongly disagree) to 7 (strongly agree). To determine the GBQ total score, the scores for the 21 items are summed up. In the present study, the German translation of the GBQ by Meyer et al. (2011) was used. In the original study by Steenbergh et al. (2002), the internal consistency (Cronbach’s alpha) was 0.9. A psychometric test of the German-language version of the GBQ is not yet available.

For the purpose of this study, survey participants who had either immigrated to Germany themselves or who were born to at least one parent who had immigrated to Germany were characterized as individuals with a migration background. In Germany, the migration background is most frequently Turkish, Eastern European, Italian, Kazakh, or Syrian. Among people with a migration background, gambling behavior varies, depending on the country of origin. Overall, however, gambling problems are more prevalent among people with a migration background than among those without (Kastirke et al., 2018).

Further items of the questionnaire (sociodemographics, type and frequency of gambling participation) also refer to the research questions formulated in the introduction. Particular attention was paid to the differentiation between three different pandemic phases: the period immediately before the first lockdown (January and February 2020), the period during the first lockdown (March–May 2020), and the period after the first lockdown (June–October 2020).

The online questionnaire was programmed using the survey software “LimeSurvey” and could be accessed and answered via URL link on mobile and desktop devices. The retrospective, cross-sectional survey started on 2 December 2020 and ended on 18 January 2021. Data preparation and statistical tests were performed using SPSS 25. In order to determine the relevance of potential influencing factors (socio-demographic characteristics, health characteristics, and gambling-related characteristics) on the change of gambling behavior during the pandemic, the calculation of univariate multinomial logistic regression analyses was performed using MPLUS 8.3 (Muthén and Muthén, 2019). Here, the metric-scaled items age, GBQ, number of higher-risk gambling forms played, and number of days gambled in the past month were centered around their means. As it can be assumed that the identified influencing factors correlate with each other (see also Table 3), both the corresponding correlations and a multivariate multinomial logistic regression were calculated (also using MPLUS) for the purpose of presenting and controlling for these dependencies.

**TABLE 1:** Parameters for changes in gambling behavior between the periods before and after the first lockdown – comparison of gambling groups (univariate multinomial logistic regression).

		Switching to higher-risk online gambling forms (*N* = 47)		Cessation of participation in higher-risk gambling forms (*N* = 302)		Adherence to higher-risk offline gambling^§^ (*N* = 263)	F/χ^2^	Significance	Total (*N* = 612)
Parameters		Mean/%	OR [95% CI]^+^	Mean/%	OR [95% CI]^+^	Mean/%			Mean/%
Gender	Male (ref. female)	87.2%	1.93 [0.78–4.78]	65.9%	**0.55 [0.38–0.80]**	77.9%	χ^2^ = 15.7	*p* < 0.001	27.3%
Age	In years	40.9 (12.0)	**0.96 [0.94–0.98]**	41.9 (13.8)	**0.97 [0.96–0.98]**	48.3 (13.8)	*F* = 17.4	*p* < 0.001	44.6 (14.1)
School education	Higher education entrance qualification/“(Fach-)Abitur” (ref. lower education)	51.1%	0.93 [0.50–1.73]	58.9%	1.28 [0.92–1.79]	52.9%	χ^2^ = 0.3	n.s.	55.7%
Migration background	Yes (ref. no)	21.3%	1.64 [0.75–3.58]	22.6%	**1.76 [1.13–2.74]**	13.8%	χ^2^ = 7.2	*p* < 0.05	18.7%
Risky drinking*	AUDIT-C: ≥5 points (ref. ≤4 points)	27.7%	0.91 [0.45–1.81]	31.1%	1.07 [0.75–1.54]	29.7%	χ^2^ = 0.3	n.s.	30.2%
Mental health*	MHI-5	68.1 (19.4)	0.99 [0.97–1.00]	70.1 (17.4)	0.99 [0.98–1.00]	72.6 (17.7)	*F* = 2.4	n.s.	71.0
Cognitive distortions*	GBQ	73.1 (27.2)	**1.03 [1.02–1.04]**	53.7% (25.3)	1.00 [0.99–1.00]	53.9% (24.1)	χ^2^ = 13.1	*p* < 0.001	55.3
Number of higher-risk gambling forms played*	Casino games, slot machine games, sports betting	1.5 (0.7)	**2.07 [1.30–3.32]**	1.2 (0.5)	1.18 [0.85–1.64]	1.2 (0.5)	*F* = 4.7	*p* < 0.01	1.2 (0.5)
Days with gambling activity*	Maximum (if multiple higher-risk gambling forms)	9.2 (8.7)	**1.06 [1.02–1.09]**	3.6 (4.8)	**0.93 [0.89–0.97]**	5.7 (6.1)	*F* = 23.3	*p* < 0.001	4.9 (6.0)
Problem gambling*	PGSI: ≥8 points (ref. ≤7 points)	27.7%	**3.09 [1.46–6.51]**	11.6%	1.06 [0.63–1.78]	11.0%	χ^2^ = 10.6	*p* < 0.01	12.6%

*^+^Values in square brackets: 95% confidence interval of the OR; values in round brackets: standard deviation of the means.*

*Referring to the time before the first lockdown (January and February 2020).

*^§^Reference group of the regression analysis: adherence to higher-risk offline gambling.*

*ORs with confidence intervals that do not include the value 1.00 are marked in bold.*

**TABLE 2:** Development of gambling behavior between the periods before and after the first lockdown.

	Switching to higher-risk online gambling forms	Cessation of participation in higher-risk gambling forms	Adherence to higher-risk offline gambling
Participation in offline higher-risk gambling forms*	**%**	**%**	**%**
All participants in offline higher-risk gambling forms (*N* = 612)	7.7% (*N* = 47)	49.3% (*N* = 302)	43.0% (*N* = 263)
Offline casino games (*N* = 195)	9.2% (*N* = 18)	64.1% (*N* = 125)	26.7% (*N* = 52)
Offline slot machine games (*N* = 339)	7.7% (*N* = 26)	49.0% (*N* = 166)	43.4% (*N* = 147)
Offline sports betting (*N* = 221)	10.9% (*N* = 24)	37.1% (*N* = 82)	52.0% (*N* = 115)

*Only gamblers who participated in higher-risk gambling forms exclusively offline before the first lockdown were included here.

**TABLE 3:** Correlations of parameters.

	Gender	Gambling problems	Higher education entrance qualification	Migration background	Risky drinking	Age	Number of higher-risk gambling forms played	Mental health	Cognitive distortions
Problem gambling	−0.114								
Higher education entrance qualification	0.063	−0.097							
Migration background	−0.003	0.071	0.099						
Risky drinking	0.007	0.016	0.047	0.038					
Age	0.188	**−0.268**	−0.075	−0.198	0.005				
Number of higher-risk gambling forms played	0.000	0.166	0.026	0.157	−0.020	−0.122			
Mental health	0.122	**−0.554**	0.051	−0.089	−0.093	0.169	−0.072		
Cognitive distortions	0.113	**0.509**	0.066	0.113	0.155	−0.148	0.170	**−0.223**	
Days with gambling activity	0.093	**0.390**	−0.130	−0.031	0.017	0.023	0.153	−0.098	**0.287**

*Tetrachoric and polychoric correlations; correlations r > 0.2 are marked bold.*

The analysis was based exclusively on anonymized data from online interviews which did not allow the authors of this publication to draw any inferences regarding the interview participants. Since there is no obligation to seek professional advice from the ethics committee for purely anonymized surveys/analyses, an ethical vote was not obtained.

## Results

As described above, only individuals who participated in higher-risk forms of gambling (casino games, slot machines, and sports betting) and had done so exclusively offline before the first lockdown, were included in the following analysis. This group of individuals had a mean age of 44.6 years and slightly more than one quarter consisted of women (see Table 1, last column). About half of the group had the highest possible school-leaving qualification and almost one fifth had either migrated themselves or were born in Germany as children of migrants. The mental health score of 71.0 – based on the period before the first lockdown – was only slightly below the average score of 75 for the German general population (Hapke et al., 2012). The proportion of those with risky drinking in the period before the first lockdown (AUDIT-C: 30.3%) also barely deviated from the findings for the general population (26%; Robert Koch-Institut, 2014).

Table 2 shows how the gambling behavior of individuals participating in higher risk gambling forms changed between the period before the first lockdown and the period following the first lockdown. After the first lockdown, less than half of these gamblers resumed offline participation in at least one of these forms of gambling and at the same time did not switch to online gambling. About half ceased participation in higher-risk forms of gambling altogether and a comparatively low 7.7% switched to corresponding online services.

Differences, however, become apparent when comparing the different forms of gambling. Only 37.1% of sports bettors had stopped engaging in higher-risk forms of gambling, whereas this was the case for 64.1% of casino gamblers. Accordingly, 52.0% of sports bettors and a notably lower 26.7% of casino gamblers adhered to their (offline) gambling activities. In contrast, only very few offline gamblers switched to online gambling services, regardless of the gambling form. In this regard, the shares range between 7.7% (slot machines) and 10.9% (sports betting).

In the following section, we will examine the factors that influence how the gambling behavior of the respondents developed over the course of the pandemic. Again, the focus of the analysis is on the three groups “Switching to higher-risk online gambling forms,” “Cessation of participation in higher-risk gambling forms,” and “Adherence to higher-risk offline gambling.” The latter group served as the reference group in subsequent regression analyses because of the continuation of their gambling behavior. Table 1 shows that no significant differences are found between these three study groups in terms of the highest qualification reached, risky drinking, and mental health.

Men had lower odds to quit gambling (instead of adhering to offline gambling), compared to women (OR = 0.55). Furthermore, individuals with higher age (OR = 0.97) and frequent participation in gambling (OR = 0.93) showed lower odds than younger and less frequent gamblers, respectively. A migration background, on the other hand, increased the odds of quitting participation in higher-risk forms of gambling (OR = 1.76).

Significant predictors for switching to higher-risk forms of online gambling (instead of adhering to offline gambling) were found in cognitive distortions (OR = 1.03), a high gambling frequency (OR = 1.06), and an existing gambling problem (OR = 3.09) (always referring to the period before the first lockdown). Older gamblers, by contrast, showed lower odds of turning to online gambling (OR = 0.96) than those of younger age.

One may assume that the factors identified as influencing a cessation of gambling or a change toward online services, are considerably intercorrelated (see also Table 3). Therefore, a multivariate multinomial logistic regression was calculated in order to control for these associations (see Table 4). In the multivariate model, female gender (OR = 0.63), younger age (OR = 0.97) and a lower frequency of gambling before the first lockdown (OR = 0.92) were found to be significant factors for quitting (instead of continuing) higher-risk offline gambling forms. Younger age, however, also constituted a relevant risk factor for switching to online gambling services (OR = 0.97). The same was true for cognitive distortions (before the first lockdown): the more severe the cognitive distortion, the higher the odds of engaging in online gambling (OR = 1.02) instead of adhering to offline services.

**TABLE 4:** Parameters for changes in gambling behavior – comparison of gambling groups (multivariate multinomial logistic regression).

	Switching to higher-risk online gambling forms (*N* = 47)		Cessation of participation in higher-risk gambling forms (*N* = 302)	
Parameters	OR^+^	95% CI^§^	OR^+^	95% CI^§^
Male (ref. female)	2.00	[0.75–5.31]	**0.63**	**[0.42–0.95]**
Age	**0.97**	**[0.95–0.99]**	**0.97**	**[0.96–0.99]**
Migration background (ref. no migration background)	1.29	[0.57–2.91]	1.53	[0.95–2.48]
Cognitive distortions (GBQ)	**1.02**	**[1.00–1.03]**	1.00	[0.99–1.01]
Number of higher-risk gambling forms played (casino games, slot machine games, and sports betting)	1.50	[0.85–2.64]	1.14	[0.80–1.62]
Days with gambling activity (maximum)	1.02	[0.98–1.07]	**0.92**	**[0.89–0.96]**
Problem gambling (PGSI: ≥8 points) (ref. ≤7 points)	1.23	[0.46–3.24]	1.24	[0.65–2.39]

*^+^Reference group: adherence to higher-risk offline gambling.*

*^§^Values in square brackets: 95% confidence interval of OR.*

*ORs with confidence intervals that do not include the value 1.00 are marked in bold.*

The contents of Tables 1, 4 give rise to the question why, e.g., gambling problems (PGSI score) or the number of gambling days (in the period before the first lockdown, respectively) were no longer statistically significant in the multivariate model. An answer to this question is given in Table 3, which shows the matrix of correlations of all the influencing factors considered above. In this context, the high correlation of the gambling problem (PGSI score) with cognitive distortions (*r* = 0.51), mental health (*r* = −0.55) and number of gambling days (*r* = 0.39) before the first lockdown, respectively, particularly stands out. All these variables were generally highly associated with one another. In the multivariate multinomial logistic regression, these intercorrelations were statistically controlled, so that in each case only one of these variables prevailed as statistically significant for quitting higher-risk gambling or for switching to online gambling.

## Discussion

According to the results of the present study, almost half of all respondents used the first lockdown to cease participation in higher-risk forms of gambling at least for a medium-term period (until the second lockdown). A Swedish study reported much lower rates of quitters of riskier forms of gambling during the first lockdown (<10%) (Månsson et al., 2021). However, these varying results may be due to the composition of the respective online panels or to specific lockdown rules.

The multivariate analysis showed that, compared to those who continued offline gambling after the lockdown, women, younger individuals, and individuals with lower gambling frequency (gambling days) were more likely to quit due to closures during the lockdown.

There are barely any studies available to compare these findings with. However, Gunstone et al. (2020) also show that female gamblers, who on average gamble less frequently than men and exhibit lower PGSI scores, were more likely than men to state that they gambled less during the lockdown because they anyhow only gambled occasionally.

Furthermore, the multivariate analysis revealed that younger gamblers as well as individuals with more pronounced cognitive distortions had an increased risk of switching to online gambling rather than adhering to offline services.

Again, it is difficult to discuss these findings in a broader research context because hardly any international research has been published on the specific research question of this study. With regard to the cessation of offline gambling during the lockdown, ambivalent results are available regarding the affected age groups: they range from younger, to middle, to older age groups (Biddle, 2020; Georgiadou et al., 2021). No comparable data are available on the other influencing factors identified in the present study.

The finding, however, that individuals with gambling problems showed a statistically significantly higher probability of switching to online gambling is confirmed (Xuereb et al., 2021). Again, no further findings are available on the other parameters identified.

There are several limitations to the present study which need to be taken into account. Members of online-access-panels are a specific population (online affine, interested in surveys, and incentive-oriented). Due to selection bias, such samples are only suitable to a limited extent for studies which claim representativeness. However, since the present study recruited specific gambling groups, selection bias is likely to be of less relevance than for representative studies targeting the general population. Nevertheless, due to the lack of current reference surveys it is difficult to assess how representative these sub-samples actually are. It should also be noted that the German-language versions of the PGSI and GBQ instruments used have not yet been validated. Furthermore, the presented results are based on retrospective (subjective) self-assessments by the respondents. Misinterpretations due to the time lag of approximately 6–9 months and social desirability cannot be ruled out. Moreover, this study only examined the short-term effects of the closure of offline gambling venues. The question whether and to what extent the described cessation and switching processes are sustainable, remains unclear.

The study provides detailed information on the expected effects of environmental prevention interventions in the gambling sector. For a considerable proportion of gamblers, the lockdown-related closure of offline gambling services with higher-risk forms of gambling apparently led to the (positive) effect of gambling cessation. This gives rise to the question as to whether short, temporary closures could be appropriate measures for providing gamblers with the opportunity to reflect on their uncontrolled gambling behavior, to reduce it, or to end it altogether. Such a claim for a “pause in supply” has also been made based on clinical observations during the pandemic (George, 2020). According to the results of the study at hand, such a “pause of supply” would not be expected to result in any significant migration of gamblers toward existing online services. If such a measure were to be implemented, addiction prevention as well as treatment and counseling services in the gambling sector would need to be prepared and equipped so that they could support potential quitters with suitable measures. According to the findings of the analyses, women, younger persons and individuals with a migration background would be the expected target groups for these measures. As we would nevertheless expect a smaller proportion of offline gamblers to turn toward online gambling in such a situation, this would also need to be considered in the process of designing adequate prevention measures, e.g., by developing campaigns which raise awareness on the risks of online gambling. Such measures would then need to be particularly targeted at younger persons, individuals with pronounced cognitive distortions, as well as frequent and problem gamblers.

Moreover, the results of the study show that in any case there is a willingness among some of the gamblers to stop engaging in higher-risk forms of gambling, at least temporarily. This finding should also be taken into account when designing and promoting self-exclusion systems.

## Data Availability Statement

The datasets generated for this study are available on request to the corresponding author.

## Ethics Statement

Ethical review and approval was not required for the study on human participants in accordance with the local legislation and institutional requirements. The patients/participants provided their written informed consent to participate in this study.

## Author Contributions

JK, CS, and SB designed the study together and organized the data collection. SB was responsible for the analyses. JK was draft of the manuscript. All authors participated in the interpretation of the data and results. HL, CS, and SB critically revised the manuscript for important intellectual content. All authors approved the final version to be published.

## Conflict of Interest

All authors have received grants for gambling research from the Federal Ministry of Health, several federal states and lottery providers.

## Publisher’s Note

All claims expressed in this article are solely those of the authors and do not necessarily represent those of their affiliated organizations, or those of the publisher, the editors and the reviewers. Any product that may be evaluated in this article, or claim that may be made by its manufacturer, is not guaranteed or endorsed by the publisher.
